# Patterns of use, effectiveness and safety of gadolinium contrast agents: a European prospective cross-sectional multicentre observational study

**DOI:** 10.1186/s12880-021-00600-9

**Published:** 2021-04-20

**Authors:** Jarl Åsbjørn Jakobsen, Carlo Cosimo Quattrocchi, Frank H. H. Müller, Olivier Outteryck, Andrés Alcázar, Wolfgang Reith, Patricia Fraga, Valeria Panebianco, Alexis Sampedro, Radoslaw Pietura

**Affiliations:** 1 Oslo University Hospital, Oslo, Norway; 2 Università Campus Bio-Medico Di Roma, Rome, Italy; 3 Radiologie Und Nuklearmedizin Ludwigshafen, Ludwigshafen, Germany; 4 Department of Neuroradiology, CHU Lille, Lille, France; 5 U1172, Lille Neuroscience and Cognition, Univ. Lille, Lille, France; 6 Fundación Jiménez-Díaz, Madrid, Spain; 7 Universitätsklinikum Des Saarlandes, Homburg, Germany; 8 Hospital Universitario del Henares, Unidad Central de Radiodiagnóstico, Coslada, Madrid, Spain; 9 Department of Radiological Sciences, Oncology and Pathology, Sapienza University/Policlinico Umberto I of Rome, Rome, Italy; 10 GE Healthcare, Madrid, Spain; 11 Department of Radiography, Medical University of Lublin, Lublin, Poland

**Keywords:** Magnetic resonance imaging, Gadolinium, Image quality, Diagnostic confidence, Adverse events

## Abstract

**Background:**

The EU gadolinium-based contrast agents (GBCA) market has changed in recent years due to the European Medicines Agency decision to suspend the marketing authorisation of linear GBCA and the marketing authorisation of new generic macrocyclic GBCA. The study aims to understand the patterns of (GBCA) use, and to study the effectiveness and safety of GBCA in routine practice across Europe.

**Methods:**

Prospective, cross-sectional, multicentre, observational study in patients undergoing contrast-enhanced magnetic resonance. Reported usage patterns included indication, referral and examination details. Assessment of effectiveness included changes in radiological diagnosis, diagnostic confidence and image quality. Safety data were collected by spontaneous patient adverse event (AE) reporting.

**Results:**

2118 patients were included from 8 centres across 5 European countries between December 2018 and November 2019. Clariscan, Dotarem (gadoteric acid), Gadovist (gadobutrol) and ProHance (gadoteridol) were utilised in 1513 (71.4%), 356 (16.8%), 237 (11.2%) and 12 (0.6%) patients, respectively. Most were performed in CNS-related indications (46.2%). Mean GBCA doses were 0.10 mmol/kg body weight, except for Gadovist (mean 0.12 mmol/kg). GBCA use increased confidence in diagnosis in 96.2% of examinations and resulted in a change in radiological diagnosis in 73.9% of patients. Image quality was considered excellent or good in 96.1% of patients and across all GBCA. Four patients reported AEs (0.19%), with only 1 (0.05%) considered serious.

**Conclusions:**

This European study confirmed that GBCAs are used appropriately in Europe for a wide range of indications. The study demonstrated a significant increase in diagnostic confidence after GBCA use and confirmed the good safety profile of GBCAs, with comparable results for all agents used.

**Supplementary Information:**

The online version contains supplementary material available at 10.1186/s12880-021-00600-9.

## Background

Contrast-enhanced magnetic resonance (CE-MR) is a widely used imaging technique crucial for several indications and diagnoses. Gadolinium-based contrast agents (GBCAs) have been used in over 450 million patients worldwide since their introduction in 1988, and are the main agents used for CE-MR [1]. GBCAs can be differentiated based on chelate chemistry, stability, ionicity, viscosity, osmolality and relaxivity, and also according to their effectiveness for specific applications [2]. Regulatory bodies recommend the use of contrast enhancement in instances wherein unenhanced MR is not sufficient to retrieve essential diagnostic information [3]. However, defining the indications that require contrast enhancement in clinical practice is often subject to local variability in factors, such as availability of resources, local protocols and expertise, patient expectations, and financial pressures and constraints. These can all affect whether the use of contrast agents is considered.

The European contrast media market has recently undergone regulatory changes due to the European Medicines Agency (EMA) decision to suspend the marketing authorisation of linear GBCAs after safety concerns, based on observations of gadolinium deposition in the brain [3]. However, the US Food and Drug Administration (FDA) has not followed suit [4]. In addition to the available macrocyclic agents, generic macrocyclic agents have entered the market including Clariscan, GE Healthcare) [5], a gadoteric acid having the same active ingredient in the same quantity and formulation as the reference product Dotarem (gadoteric acid; Guerbet Laboratories) [5, 6].

To our knowledge, no prospective study mapping the real-world use of GBCAs has been performed across all GBCAs, indications and European populations since the EMA suspended the marketing authorisation of linear agents in Europe. Considering this environment, it is important to understand how clinicians currently use GBCAs in clinical practice. This includes referral patterns, indications, GBCA selection and dosing, how contrast enhancement can contribute to a confident diagnosis and real-world safety.

This study (NCT03455283; EUPAS21473) aimed to explore: the patterns of GBCA use, by assessing indications and dosages; and effectiveness and safety of GBCA use, by assessing image quality, diagnostic confidence and adverse events (AEs).

## Methods

### Study design

This cross-sectional, multicentre, observational study with prospective recruitment was performed in patients scheduled for gadolinium contrast-enhanced magnetic resonance (CE-MR) examinations as part of their normal clinical workup.

Centre eligibility required an independent decision of inclusion of Clariscan in the formulary for CE-MR examinations and the availability of cumulative electronic patient data to enable cumulative data reporting at study end. A single radiological team, under the supervision of a single Principal Investigator per centre, was allowed to participate. Eligible patients included males and females of all age groups and pathologies who required CE-MR imaging as part of their diagnostic workup, and whose physician or radiologist had made the decision to use extracellular GBCAs during routine clinical practice. Eligible patients provided written informed consent prior to inclusion.

### Population

No sample size calculation was performed, owing to the observational nature of the study, and no formal hypothesis was tested. A minimum 3-months recruitment period and 50 patients per centre were targeted to ensure multicentre representation.

Eight centres across five European countries (France, Germany, Italy, Poland and Spain) participated. Consenting patients undergoing CE-MR examinations between December 2018 and November 2019 were included.

### Procedures

No medical procedures in addition to those planned as part of the standard clinical routine were performed. Patient data related to CE-MR examinations were transferred and stored for further analysis in a secure and EU/EEA-compliant database. Ethics and regulatory approvals were obtained according to local regulations.

### Variables

The data collected included patient demographics, relevant medical history and medications, referral details, working diagnosis, organ/body region indication, MR examination details (including injection volumes and dosing), scan and MR protocol parameters, and source of funding.

The assessment of effectiveness included changes in radiological diagnosis (yes/no), diagnostic confidence ratings and CE-MR image quality. Diagnostic confidence was assessed by the local radiologist on a 0–100 percent scale, performed twice. A confidence assessment was performed for the non-enhanced images at baseline and following the CE-MR for the enhanced images. Image quality was reported on a 4-point scale based on previously described scales for MRI and MRA [7, 8]. Spontaneously reported patient AEs were documented, and classified in terms of severity, relation to the GBCA, course of treatment and latency (immediate: < 1-h post-injection; delayed: 1 h to 7 days post-injection). AEs were summarised using the current MedDRA coding system.

### Statistics

Statistical analysis was performed using SAS Software Version 9.4. Descriptive analysis was complemented by explorative statistical tests (ANOVA for continuous endpoints, Chi-Square tests for categorical endpoints) at an alpha of 0.05 and after stratification by centre, specialty, country, GBCA type, BMI, age, referral quality, history of allergy and AE occurrence, where applicable.

## Results

### Population

#### Baseline demographics

A total of 2149 patients were screened, of which 31 were excluded (12 patients due to lack of consent and 19 due to incomplete data). A total of 2118 patients were analysed. Most patients were adults, with 1191 patients (56.2%) aged 19–59 years and 915 patients (43.2%) aged ≥ 60 years. Most patients were female (n = 1261; 59.5%). The mean body mass index (BMI) was 25.9 kg/m^2^ (standard deviation [SD] 15.6–58.6); 927 (43.8%) had normal BMI, 810 (38.2%) were overweight, 340 (16.1%) were obese and 41 (1.9%) were underweight (Table 1). There were no significant differences in demographic variables among the different GBCAs used (data not shown).

**Table 1:** Demographic data and baseline characteristics of study population

Subjects recruited, N (%)	2118 (100)
Country, n (%)	
Poland	770 (36.4)
Italy	547 (25.8)
Germany	427 (20.2)
Spain	219 (10.3)
France	155 (7.3)
Sex, n (%)	
Female	1261 (59.5)
Male	857 (40.5)
Age, years, mean (SD)	55 (15.8)
Age category, n (%)	
0–18 years	12 (0.6)
19–59 years	1191 (56.2)
≥ 60 years	915 (43.2)
Height, meters, mean (SD)	1.7 (0.1)
Weight, kg, mean (SD)	73.7 (15.4)
BMI, kg/m^2^, mean (SD)	25.9 (4.7)
BMI category, n (%)	
Underweight (BMI < 18.5)	41 (1.9)
Normal (BMI ≥ 18.5–< 25)	927 (43.8)
Overweight (BMI ≥ 25–< 30)	810 (38.2)
Obese (BMI ≥ 30)	340 (16.1)
Comorbidity, n (%)	
Hypertension	350 (16.5)
Allergy	159 (7.5)
Previous allergy to iodine media	12 (0.6)
Previous allergy to GBCA	2 (0.1)
Malignancy/cancer	140 (6.6)
Diabetes mellitus	114 (5.4)
Neurological symptom	76 (3.6)
Heart failure	26 (1.2)
Autoimmune disease	25 (1.2)
Renal impairment	16 (0.8)
eGFR < 30 mL/min/m^2^	2 (0.1)
Hepatic impairment	9 (0.4)
Other	80 (3.8)
Premedication, n (%)	
Steroids	17 (0.8)
Antihistamines	12 (0.6)
Concomitant medications, n (%)	
Anti-hypertensives	309 (14.6)
Chemotherapy	102 (4.8)
Antidiabetic drugs	94 (4.4)
Other reported	225 (10.6)

*BMI* body mass index, *eGFR* estimated glomerular filtration rate, *GBCA* gadolinium-based contrast agent, *SD* standard deviation

#### Patient history and medications

Comorbidities were reported in 998 patients (47.1%). The most common, which occurred in more than 5% of patients, was hypertension (n = 350; 16.5%) followed by history of allergic conditions (n = 159; 7.5%), cancer (n = 140; 6.6%) and diabetes (n = 114; 5.4%). Renal impairment was reported in 16 patients (0.8%), whereas hepatic impairment was seen in 9 patients (0.4%). Previous allergy to iodinated contrast media was reported in 12 patients (0.6%) and to other GBCAs in 2 patients (0.1%). Steroids and antihistamines were administered as pre-medication in 17 (0.8%) and 12 (0.6%) patients, respectively. Concomitant medications were reported in 730 (34.5%) patients, mostly anti-hypertensives (n = 309; 14.6%), chemotherapy (n = 102; 4.8%) and antidiabetic drugs (n = 94; 4.4%) (Table 1).

### Pattern and quality of referral and type of reimbursement

In this study, 42 patients (2%) were involved in emergency procedures requiring CM administration, 1356 (64%) were referred for routine diagnosis and 720 (34%) were undergoing follow-up procedures for a known disease. Referral information was considered insufficient in 101 (4.8%), satisfactory in 1768 (83.5%) and well detailed with a clear medical question in 249 patients (11.8%). The costs of the procedure and GBCA were generally reimbursed (n = 1816; 95.4%), mostly by the state (n = 1274; 70.2%) or private insurance (n = 459; 25.3%).

### CE-MR examination and GBCA administration

#### Body regions examined and scan type

The majority of CE-MR examinations were performed for CNS-related indications (n = 902; 42.6%), followed by indications related to breast (n = 242; 11.4%), musculoskeletal (n = 237; 11.2%), head and neck (n = 132; 6.2%), urinary tract including bladder (n = 103; 4.9%) and hepatobiliary (n = 100; 4.7%) (Table 2) with some variations between countries (Additional file 1: Table S1). The majority of examinations were MRI (n = 1989; 93.9%), whereas MRA was used in 129 (6.1%) procedures (either as angiography only or as a combined MRI/MRA procedure).

**Table 2:** Type of contrast, procedures and indications

Type of contrast used	Total^a^	Clariscan	Dotarem	Gadovist
n (%)	2118 (100)	1513 (71.4)	356 (16.8)	237 (11.2)
Type of indication, n (%)				
CNS	902 (42.6)	597 (39.5)	169 (47.5)	130 (54.9)
Organ/whole body	1186 (56.0)	900 (59.5)	181 (50.8)	99 (41.8)
Angiography indication	30 (1.4)	16 (1.1)	6 (1.7)	8 (3.4)
Type of procedure, n (%)				
MRI	1989 (93.9)	1427 (94.3)	345 (96.9)	208 (87.8)
CNS	811 (38.3)	529 (35.0)	165 (46.3)	113 (47.7)
Organ/whole body	1178 (55.6)	898 (59.4)	180 (50.6)	95 (40.1)
MRA^b^	129 (6.1)	86 (5.7)	11 (3.1)	29 (12.2)
Organ under examination, n (%)				
Brain/meninges/spinal cord	902 (42.6)	597 (39.5)	169 (47.5)	130 (54.9)
Breast	242 (11.4)	178 (11.8)	43 (12.1)	18 (7.6)
Musculoskeletal system	237 (11.2)	214 (14.1)	14 (3.9)	9 (3.8)
Head and neck	132 (6.2)	94 (6.2)	19 (5.3)	16 (6.8)
Urinary tract including bladder	103 (4.9)	93 (6.1)	8 (2.2)	2 (0.8)
Hepatobiliary	100 (4.7)	63 (4.2)	26 (7.3)	11 (4.6)
Genital tract including gonads	63 (3.0)	34 (2.2)	12 (3.4)	17 (7.2)
Gastrointestinal tract	56 (2.6)	41 (2.7)	10 (2.8)	5 (2.1)
Pancreas	35 (1.7)	27 (1.8)	6 (1.7)	2 (0.8)
Cardiovascular	24 (1.1)	11 (0.7)	9 (2.5)	4 (1.7)
Renal	17 (0.8)	16 (1.1)	1 (0.3)	
Endocrine glands	16 (0.8)	12 (0.8)	4 (1.1)	
Other	191 (9.0)	133 (8.8)	35 (9.8)	23 (9.7)

*CNS* central nervous system, *MRA* magnetic resonance angiography, *MRI* magnetic resonance imaging, *SD* standard deviation

^a^Data for ProHance are not shown separately owing to the low number of patients (n = 12 [0.6%])

^b^MRA type of procedures include procedures as angiography only and combined MRI/MRA procedures

#### Type of GBCA and injection details

Gadoteric acid was used in the majority of patients, with use of Clariscan in 1513 patients (71.4%) and Dotarem in 356 patients (16.8%), followed by gadobutrol (Gadovist) in 237 patients (11.2%) (Table 3). Although the protocol allowed for the use of any macrocyclic GBCA, gadoteridol (ProHance) was only used in 12 patients (0.6%).

**Table 3:** Injections details by GBCA^a^

	Total^a^ (N = 2118)	Clariscan (n = 1513)	Dotarem (n = 356)	Gadovist (n = 237)
Mean volume, mL, mean	14.2 (3.4)	15.0 (2.6)	14.2 (3.2)	8.5 (2.9)
Mean dose, mmol/kg, mean (SD)	0.10 (0.02)	0.10 (0.02)	0.10 (0.02)	0.12 (0.04)
Mean dose by type of procedure, mmol/kg, mean (SD)				
MRI	0.10 (0.02)	0.10 (0.02)	0.10 (0.02)	0.11 (0.02)
CNS	0.10 (0.02)	0.10 (0.01)	0.10 (0.01)	0.11 (0.02)
Organ/whole body	0.10 (0.02)	0.10 (0.02)	0.10 (0.02)	0.11 (0.03)
MRA^b^	0.13 (0.04)	0.11 (0.03)	0.13 (0.04)	0.18 (0.04)
Dose category in mmol/kg, n (%)				
≤ 0.1	1182 (55.8)	887 (58.6)	208 (58.4)	80 (33.8)
> 0.1–0.2	919 (43.4)	623 (41.2)	147 (41.3)	144 (60.8)
> 0.2–0.3	17 (0.8)	3 (0.2)	1 (0.3)	13 (5.5)
Injector use, n (%)	1362 (64.3)	998 (66.0)	209 (58.7)	146 (61.6)
Subsequent saline injection, n (%)	1461 (69.0)	1032 (68.2)	253 (71.1)	167 (70.5)
Saline injection volume, mL, mean (SD)	17.2 (8.3)	15.6 (8.6)	21.9 (7.9)	19.8 (1.7)
Field strength				
1.5 T	1909 (90.1)	1391 (91.9)	311 (87.4)	207 (87.3)
3 T	208 (9.8)	122 (8.1)	45 (12.6)	29 (12.2)
> 3 T	1 (0.0)	0 (0.0)	0 (0.0)	1 (0.4)

*CNS* central nervous system, *GBCA* gadolinium-based contrast agent, *MRA* magnetic resonance angiography, *MRI* magnetic resonance imaging, *SD* standard deviation

^a^Data for ProHance are not shown separately owing to the low number of patients (n = 12 [0.6%])

^b^MRA type of procedures include procedures as angiography only and combined MRI/MRA procedures

The mean (SD) GBCA dose was 0.10 mmol/kg bodyweight (0.02) and the maximum administered dose was 0.3 mmol/kg. Dosing was within the recommended dose of ≤ 0.1 mmol/kg in 1182 patients (55.8%). A total of 919 patients (43.4%) received doses that were within the range of > 0.1–0.2 mmol/kg, while 17 patients (0.8%) received doses in the range of > 0.2–0.3 mmol/kg. The mean dose was 0.10 mmol/kg for all products, except Gadovist which was administered at a significantly higher mean (SD) dose (0.12 mmol/kg [0.02]; p < 0.001) than the other GBCAs. Gadovist administrations were in the range of > 0.1–0.2 mmol/kg in 144 patients (60.8%) and > 0.2–0.3 mmol/kg in 13 patients (5.5%) (Table 3).

#### Field strength

1.5 T MRI was used in the majority of the studies (n = 1909, 90.1%), 3 T less frequently (n = 208, 9.8%) or > 3 T (n = 1, 0.0%). Majority of Clariscan (n = 1391 (91.9%)), Dotarem (n = 311 (87.4%)) and Gadovist (n = 207 (87.3%)) studies were performed using 1.5 T MRI (Table 3).

### Effectiveness

#### Change in radiological diagnosis, increase in confidence in diagnosis

**Fig. 1:**
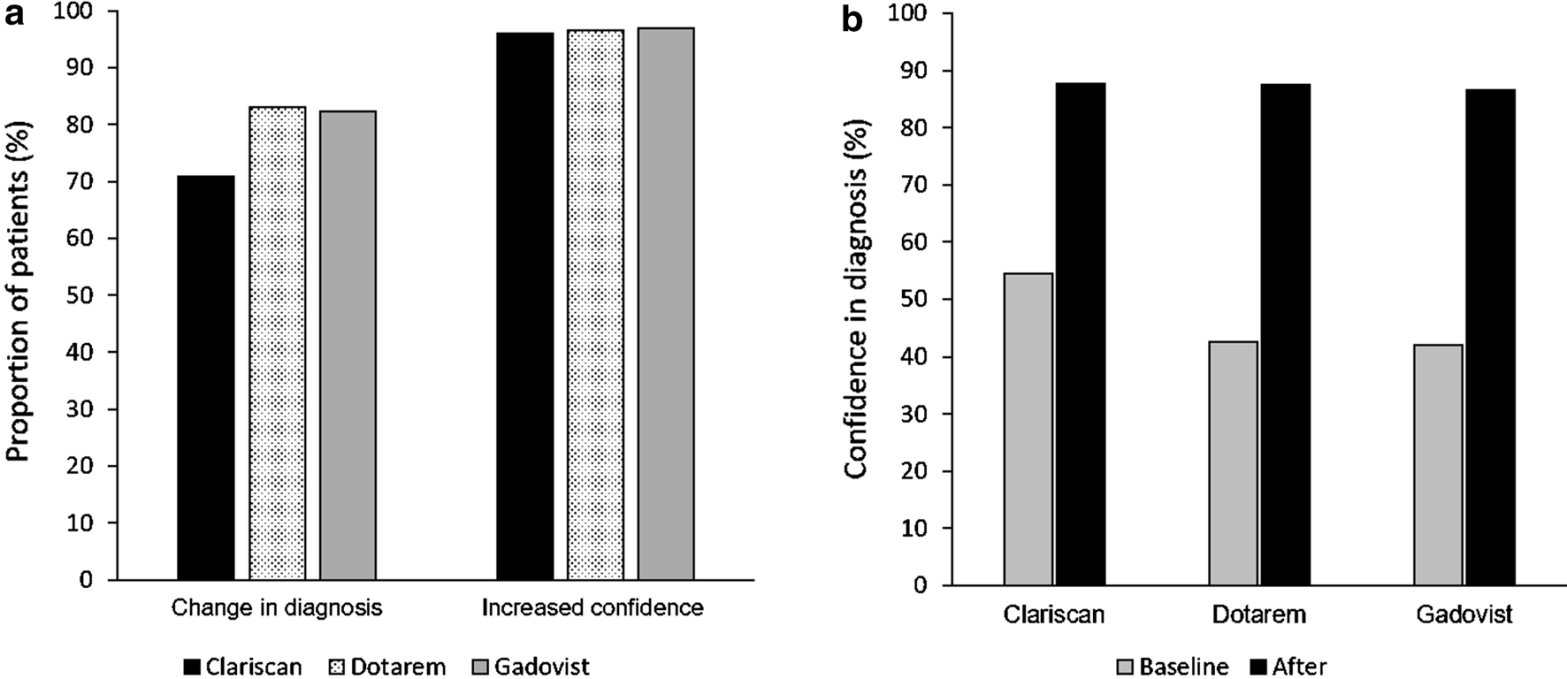
Changes in radiological diagnosis (**a**) and confidence (**a**, **b**) after CE-MRI/MRA. *CE* contrast-enhanced, *MRI* magnetic resonance imaging, *MRA* magnetic resonance angiography

The use of CE-MR procedure increased confidence in diagnosis in 2037 examinations (96.2%). Confidence in diagnosis increased from 51.1 at baseline to a mean post-contrast confidence of 87.6 (measured on a 0–100 scale) and resulted in a change in the radiological diagnosis in 73.9% of patients (Fig. 1; Table 4).

**Table 4:** Changes in radiological diagnosis and confidence after CE-MRI/MRA^a^

	Total (N = 2118)	Clariscan (n = 1513)	Dotarem (n = 356)	Gadovist (n = 237)
Changes in radiological diagnosis, n (%)				
Yes	1566 (73.9)	1071 (70.8)	296 (83.1)	195 (82.3)
No	552 (26.1)	442 (29.2)	60 (16.9)	42 (17.7)
Increased confidence in diagnosis, n (%)				
Yes	2037 (96.2)	1453 (96.0)	344 (96.6)	230 (97.0)
No	81 (3.8)	60 (4.0)	12 (3.4)	7 (3.0)
Confidence before CE-MR (0–100), mean (SD)	51.1 (23.7)	54.5 (23.3)	42.6 (23.7)	42.0 (19.8)
Confidence after CE-MR (0–100), mean (SD)	87.6 (12.6)	87.7 (13.2)	87.5 (9.9)	86.6 (12.2)

*CE* contrast-enhanced, *MR* magnetic resonance, *MRA* magnetic resonance angiography, *MRI* magnetic resonance imaging, *SD* standard deviation

^a^Data for ProHance are not shown separately owing to the low number of patients (n = 12 [0.6%])

#### Image quality

**Fig. 2:**
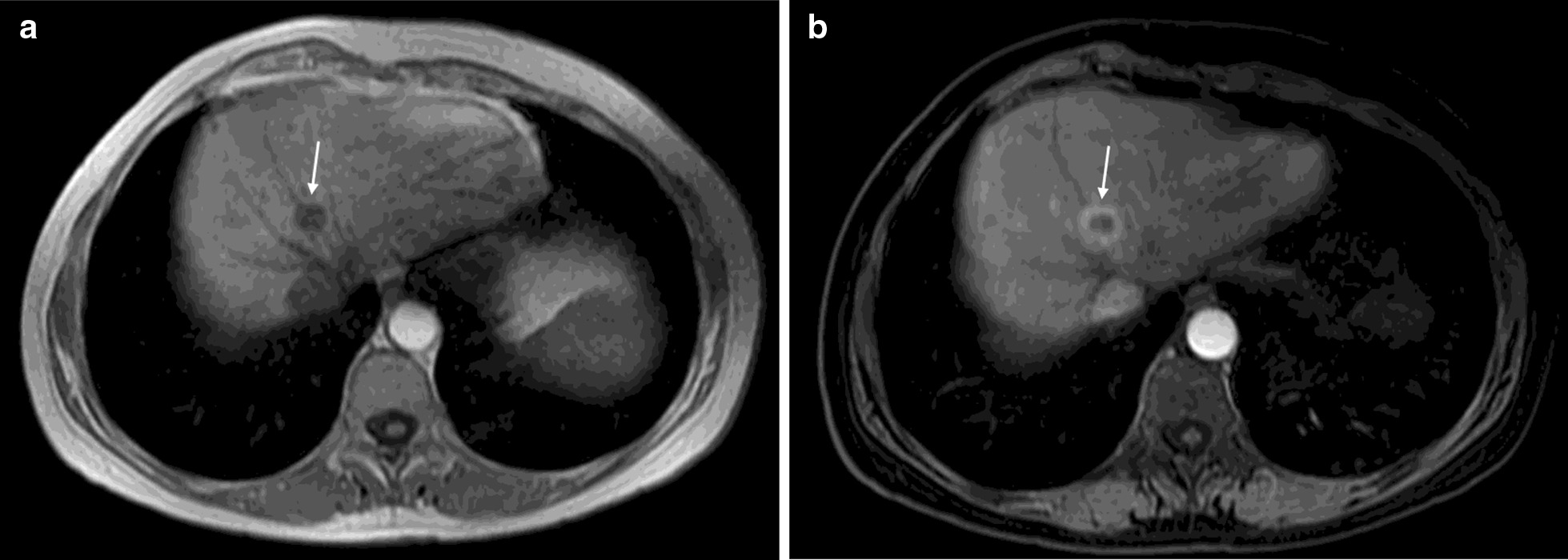
Liver lesion pre and post administration of contrast. Liver lesion pre and post administration of contrast. Middle-aged woman (45–65 year old category) with a history of breast cancer, treated with mastectomy and chemotherapy in 2001 and ovarian cancer in 2011 treated with surgery (hysterectomy and double adnexectomy). **a** The follow-up study in 2019 identifies a hypointense liver lesion (arrow) in T1 without contrast and **b** after the administration of contrast (Clariscan, 0.10 mmol/kg), in the arterial phase of the dynamic study, shows peripheral uptake. After partial hepatectomy, liver metastasis of high-grade ovarian serous-papillary carcinoma is confirmed. Image quality was reported as good by site investigator. Images from Hospital Universitario del Henares, Spain

**Fig. 3:**
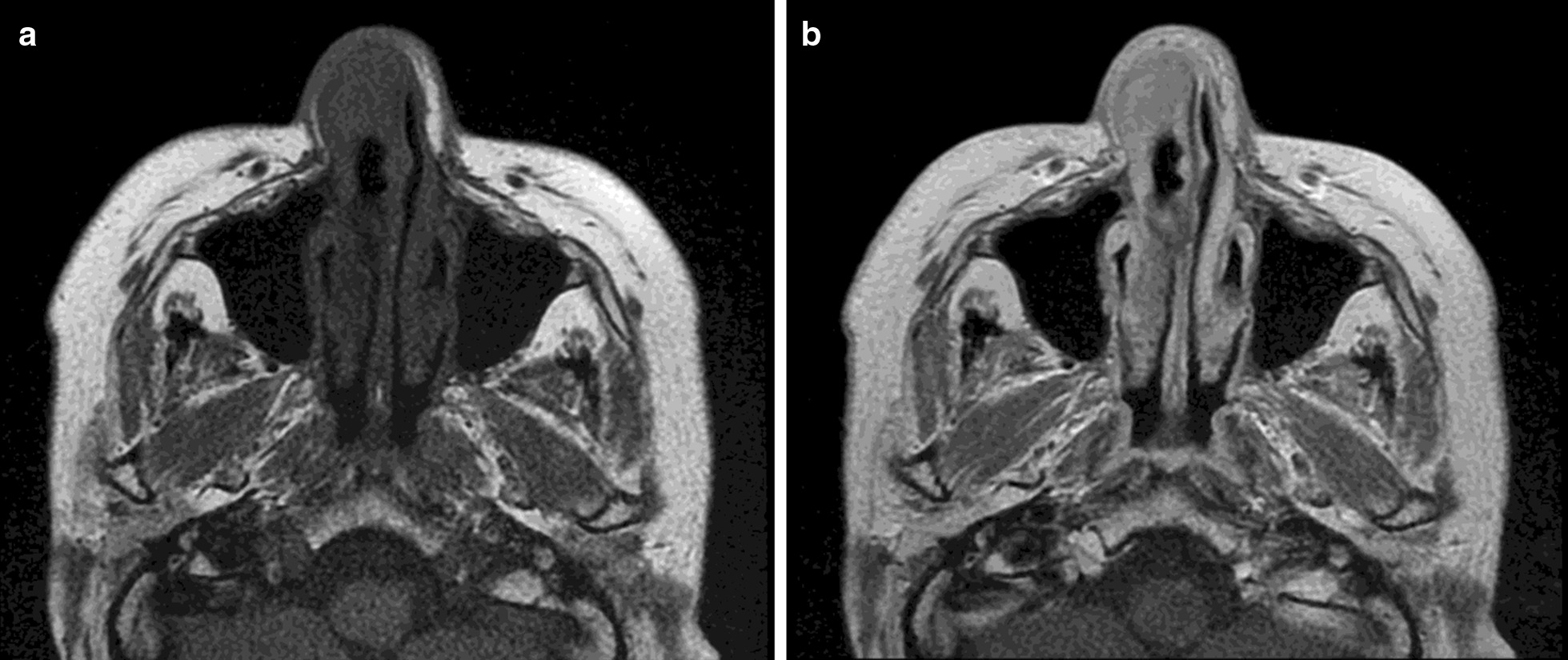
Nasal tumor pre and post administration of contrast. Nasal tumor pre and post administration of contrast. Elder woman (≥ 75 year old category). Tumor extension study in right nasal dorsum after skin biopsy corresponding to sebaceous cell carcinoma. **a** Hypointense lesion in T1 without contrast, poorly defined. **b** After administration of contrast (Clariscan, 0.10 mmol/kg), it shows homogeneous enhancement and allows to distinctly define the border of the right nasal cavity and nasal septum, without extension in depth. Image quality was reported as good by site investigator. Images from Hospital Universitario del Henares, Spain

Overall, image quality was considered excellent or good in 2035 patients (96.1%); 959 (45.3%) were excellent, 1076 (50.8%) were good, 78 (3.7%) were fair and 5 (0.2%) were poor (Table 5). There was no significant difference in the quality of images with generic gadoteric acid versus other CE-MR images (*p* = 0.7044). Figures 2 and 3 show examples of CE-MR liver and nasal cavity examination, collected as part of the study before (2a, 3a) and after (2b, 3b) the use of a GBCA at 1.5 T.

**Table 5:** Image quality by GBCA^a^

*Image quality (4 points scale)*	Total (N = 2118)	Clariscan (n = 1513)	Dotarem (n = 356)	Gadovist (n = 237)
Excellent, n (%)	959 (45.3)	695 (45.9)	178 (50.0)	84 (35.4)
Good, n (%)	1076 (50.8)	757 (50.0)	172 (48.3)	137 (57.8)
Fair, n (%)	78 (3.7)	57 (3.8)	6 (1.7)	15 (6.3)
Poor, n (%)	5 (0.2)	4 (0.3)	0 (0.0)	1 (0.4)

*GBCA* gadolinium-based contrast agent

^a^Data for ProHance are not shown separately owing to the low number of patients (n = 12 [0.6%])

The range of image quality assessment differed among centres. For example, one centre considered image quality to be excellent in 0.4% of patients, while another considered it to be excellent in 98.3%; quality was considered good in 1.7–96.0% of patients, fair in 0–32.0% and poor in 0–0.8%.

**Fig. 4:**
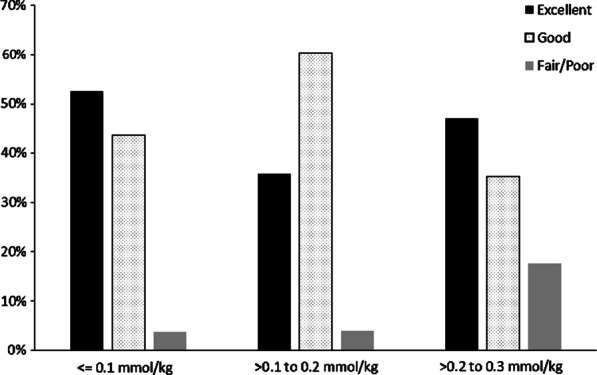
Quality of the image rating by dose category

GBCA doses higher than the recommended (0.1 mmol/kg) did not result in a statistically significant increase in image quality. In fact, there was a trend towards a higher proportion of excellent images with the lower dose (Fig. 4).

### Safety

Nine AEs were reported in 4 patients (0.19%). All 4 reports were associated with the administration of Clariscan and were considered to have a reasonable causal relationship with the CM. The events were nonserious in 3 patients (nausea, presyncope, pruritus/urticaria) and serious in 1 patient (0.05%)—this was a 27-year-old male who experienced a severe hypersensitivity reaction with symptoms of hypotension, nausea, pruritus, rash and sneezing. The patient was treated with antihistamines and recovered within 2 h.

No formal comparison between the GBCAs used was possible due to the size of the study and the low overall number of AEs reported.

## Discussion

This was the first prospective observational study of GBCA usage across Europe since the EMA decision to suspend the marketing authorisation of linear agents and introduction of the generic macrocyclic GBCA Clariscan. After the inclusion of more than 2000 subjects across 5 countries, the study demonstrated that the current clinical use of GBCA was well aligned with recommendations from the corresponding Summary of Product Characteristics (SmPC), and that GBCA use had a significant impact on CE-MRs by improving the diagnostic confidence of the radiologists in more than 95% of patients, inducing a change in radiological diagnosis in more than 70% of the examinations performed. The overall rate of AEs in 0.2% of patients (< 0.1% being serious) was low and comparable with rates reported in the literature; however, due to the limited study size, the use of different GBCAs in the participating centres and overall low AE rates, no formal safety comparison between the GBCAs could be performed.

Recent EU regulatory changes have restricted the use of GBCAs to macrocyclic compounds in cases wherein essential diagnostic information cannot be obtained with unenhanced scans, with the requirement that the lowest dose that provides sufficient enhancement for diagnosis is used [3]. Clariscan was approved by the EMA, and more recently by the FDA, on the basis of equal formulation (active ingredient and excipients) and quality, and subsequently assumed similar efficacy and safety as the reference gadoteric acid based GBCA (Dotarem), which has been available for clinical use since 1989 [6].

Due to the broad range of patients included in the study, patient demographics and medical history varied. Notes from the referring physicians were found to vary in detail and accuracy, demonstrating room for improvement in how data are recorded in clinical practice. As previously reported, accurate referral notes are key to ensure that radiologists perform the appropriate examination, including GBCA use and parameters [9]. Additionally, paucity of details regarding patient history and medications can negatively impact patient safety when alternative sources of data to the referral notes are not available [10].

This study highlighted differences in the patterns of use of GBCAs. These differences appear to be related to individual characteristics of the centres involved and their radiology units. Differences in the type of examination, doses, use of injectors and saline were mostly driven by differences in the routine practice of the participating centres.

Regarding the GBCA doses and volumes injected, it is apparent that all GBCAs were mostly used according to the SmPC posology and EU recommendations to use the minimum dose (i.e. 0.1 mmol/kg). The mean dose for Clariscan and Dotarem in this study was 0.1 mmol/kg, whereas Gadovist was used more often at a higher dose (mean 0.12 mmol/kg). One possible explanation for this difference might be the use of different doses for different clinical indications; Gadovist was used more often at standard volumes rather than body weight-adapted volumes, resulting in slightly higher doses per kg of body weight across the indications compared with Clariscan and Dotarem. In addition, the fact that the proportion of angiographies was higher in the Gadovist group (12.2%) than Clariscan (5.7%) or Dotarem (3.1%) may have also contributed to the higher doses observed with this product.

Regarding image quality, we acknowledge that differences in the relaxivity of GBCAs based on their structure are a frequent subject of discussion. Relaxivity of macrocyclic agents at 1.5 T measured in water/plasma ranges from 2.9/3.6 L/mmol/s for gadoteric acid (Clariscan, Dotarem) to 3.3/5.2 L/mmol/s for gadobutrol (Gadovist) [11]. However, despite this difference in relaxivity, the overall image quality and confidence in diagnosis with Gadovist did not appear to differ from other GBCA. This observation supports findings from the REMIND study, a prospective, randomised, cross-over trial that demonstrated non-inferiority of gadoteric acid versus gadobutrol for lesion visualisation and characterisation of brain tumours after administration of similar doses (0.1 mmol/kg body weight) [7].

The increases in the diagnostic confidence and the changes in radiological diagnosis as a result of appropriate GBCA use indicates their benefit in this setting for most patients. The image quality was good or excellent in 96.1% of the procedures, without significant differences among the different types of GBCAs. The excellent or good images (96%) with the generic GBCA support the assumption of its comparability with other GBCAs used in this study with regard to efficacy. This is in line with previous studies using gadoteric acid (Dotarem) showing good or excellent image quality in 85.8–97.5% of patients [12–15].

Nine AEs were reported in 4 patients (0.19%) during the study, with one (< 0.1%) serious case, all of which were reported after Clariscan use. All 4 cases were reported in 3 centres with high (98.1%) Clariscan use and the overall incidence of AEs was low; therefore, no formal comparison between GBCAs could be made in terms of AE rates. Furthermore, the incidence of AEs in this study is comparable with the incidence observed in other prospective post-authorisation studies and registries with various GBCAs in different indications. In a major European prospective registry, promoted by the European Society of Cardiovascular Radiology, with 72,839 GBCA-enhanced cardiac MRs, Uhlig et al. found a total incidence of AEs of 0.36% and severe AEs of 0.03% [16]. In this study, gadoteric acid had the lowest incidence of AEs (odds ratio [OR] 0.89) with non-significant difference versus gadobutrol (reference OR 1), whereas gadoteridol had a significantly higher incidence of AEs than gadobutrol (OR 3.58). In a retrospective study of hospital data on reported allergic reactions to GBCA in 147,624 patients, Sodagari et al. found an overall rate of reactions of 0.17%, with < 0.01% evaluated as severe [17]. Regarding individual agent prospective studies with gadoteric acid, Soyer et al. reported an AE incidence of 0.12% (0.03% serious AEs), and Maurer et al. reported an AE rate of 0.34% (< 0.01% serious AEs) in 84,621 patients [13, 18]. For gadobutrol, Power et al. reported an incidence of 0.32% allergic-like reactions in 32,991 patients, and for gadoteridol, Morgan et al. found an overall AE rate of 0.67% and 0.01% severe in 28,078 patients [19, 20]. Considering these AE rates, the introduction of Clariscan as a new brand of gadoteric acid does not seem to be associated with an increased rate of AEs or a potential Weber effect [21].

This study has several limitations. As with any registry study, the range of participating centres represents a limited sample of the European MR clinical landscape and only provides data representative of a specific time period within the participating centres. Nevertheless, efforts were made to capture a diverse range of public and private clinics in different parts of Europe and with different patient volumes and speciality focusses.

Only centres that had Clariscan on shelf were permitted to participate but there were no restrictions on the use of other GBCAs as part of hospital practice and in contrast to previously published PMS studies that included only one GBCA. This resulted in a high use of Clariscan which may in part be due to participation of 3 centres that only used Clariscan for CE-MR. With 71.4%, 16.8%, 11.2%, and 0.6% use of Clariscan, Dotarem, Gadovist or ProHance the data allowed to show a wider picture of the usage patterns, but the results do not allow a reliable comparison between the individual agents.

This study was observational, and many of the observations may be biased by factors that cannot be properly controlled for in a univariate descriptive analysis. Furthermore, AE reporting could have been underestimated, as reporting could only happen spontaneously once the patient left the radiology department. Although patients were encouraged to report any events, this may have reduced the overall reported AE rate. However, it is unlikely to impact the reported rate of serious AEs, which mostly occur as immediate reactions.

## Conclusions

In summary, this study confirmed an appropriate use of GBCAs in Europe for a wide range of indications, mostly within recommended doses. GBCA resulted in excellent to good image quality with a subsequent improvement in diagnostic confidence in more than 96% of patients. This resulted in a change in diagnosis in more than 70% of patients. The previously known good safety profile of the GBCAs used was confirmed, including the more recently introduced generic gadoteric acid Clariscan.

## Supplementary Information

**Additional file 1: Table S1**. Organ under examination, total and by country.

## Data Availability

The datasets used and/or analyzed during the current study are available from the corresponding author on reasonable request.

## Abbreviations

AE: Adverse event
BMI: Body mass index
CE-MR: Contrast-enhanced magnetic resonance
GBCA: Gadolinium-based contrast agent
EMA: European Medicines Agency
EU: European Union
MRA: Magnetic resonance angiography
SAE: Serious adverse event
