# Supplementary material for: Patterns of use, effectiveness and safety of gadolinium contrast agents: a European prospective cross-sectional multicentre observational study
Source: BMC Med Imaging. 2021 Apr 20;21:74. doi: 10.1186/s12880-021-00600-9 (PMC8056663; doi:10.1186/s12880-021-00600-9)
Supplement: Supplementary file 1 — Additional file 1: Table S1. Organ under examination, total and by country. [file 12880_2021_600_MOESM1_ESM.docx]

Table S1 Organ under examination, stratified by country

| **Organ under examination, n (%)** | **Total (N=2118)** | | **Poland (n=770)** | | **Italy**  **(n=547)** | | **Germany (n=427)** | | **France (n=155)** | | **Spain (n=219)** | |
| --- | --- | --- | --- | --- | --- | --- | --- | --- | --- | --- | --- | --- |
| Brain/meninges/spinal cord | 902 | (42.6) | 348 | (45.2) | 135 | (24.7) | 207 | (48.5) | 142 | (91.6) | 70 | (32.0) |
| Breast | 242 | (11.4) | 115 | (14.9) | 93 | (17.0) |  |  |  |  | 34 | (15.5) |
| Musculoskeletal system, including bones, joints and muscles | 237 | (11.2) | 19 | (2.5) | 43 | (7.9) | 167 | (39.1) |  |  | 8 | (3.7) |
| Other | 189 | (8.9) | 117 | (15.2) | 51 | (9.3) | 15 | (3.5) |  |  | 6 | (2.7) |
| Head and neck | 132 | (6.2) | 41 | (5.3) | 41 | (7.5) | 21 | (4.9) | 10 | (6.5) | 19 | (8.7) |
| Urinary tract including bladder | 103 | (4.9) | 8 | (1.0) | 85 | (15.5) | 2 | (0.5) |  |  | 8 | (3.7) |
| Hepatobiliary | 100 | (4.7) | 53 | (6.9) | 17 | (3.1) | 7 | (1.6) |  |  | 23 | (10.5) |
| Genital tract including gonads | 63 | (3.0) | 29 | (3.8) | 24 | (4.4) |  |  |  |  | 10 | (4.6) |
| Gastrointestinal tract | 56 | (2.6) | 19 | (2.5) | 19 | (3.5) | 3 | (0.7) |  |  | 15 | (6.8) |
| Pancreas | 35 | (1.7) | 13 | (1.7) | 15 | (2.7) | 1 | (0.2) |  |  | 6 | (2.7) |
| Cardiovascular | 24 | (1.1) | 1 | (0.1) | 14 | (2.6) |  |  |  |  | 9 | (4.1) |
| Renal | 17 | (0.8) | 2 | (0.3) | 7 | (1.3) | 4 | (0.9) |  |  | 4 | (1.8) |
| Endocrine glands | 16 | (0.8) | 5 | (0.6) | 2 | (0.4) |  |  | 3 | (1.9) | 6 | (2.7) |
| Whole body | 1 | (<0.1) |  |  |  |  |  |  |  |  | 1 | (0.5) |
| Bronchial tissue/lungs | 1 | (<0.1) |  |  | 1 | (0.2) |  |  |  |  |  |  |

Empty cells indicate a value of zero
